# Estimations of the lethal and exposure doses for representative methanol symptoms in humans

**DOI:** 10.1186/s40557-017-0197-5

**Published:** 2017-10-02

**Authors:** Chan-Seok Moon

**Affiliations:** Department of Industrial Health, Catholic University of Pusan, #57, Oryundae-ro, Geumjeong-gu, Busan, 46252 South Korea

**Keywords:** Methanol, Lethal dose, Blindness, Dose-response relation, Exposure, Review

## Abstract

**Background:**

The aim of this review was to estimate the lethal and exposure doses of a representative symptom (blindness) of methanol exposure in humans by reviewing data from previous articles.

**Methods:**

Available articles published from 1970 to 2016 that investigated the dose-response relationship for methanol exposure (i.e., the exposure concentration and the biological markers/clinical symptoms) were evaluated; the MEDLINE and RISS (Korean search engine) databases were searched. The available data from these articles were carefully selected to estimate the range and median of a lethal human dose. The regression equation and correlation coefficient (between the exposure level and urinary methanol concentration as a biological exposure marker) were assumed from the previous data.

**Results:**

The lethal human dose of pure methanol was estimated at 15.8–474 g/person as a range and as 56.2 g/person as the median. The dose-response relationship between methanol vapor in ambient air and urinary methanol concentrations was thought to be correlated. An oral intake of 3.16–11.85 g/person of pure methanol could cause blindness. The lethal dose from respiratory intake was reported to be 4000–13,000 mg/l. The initial concentration of optic neuritis and blindness were shown to be 228.5 and 1103 mg/l, respectively, for a 12-h exposure.

**Conclusion:**

The concentration of biological exposure indices and clinical symptoms for methanol exposure might have a dose-response relationship according to previous articles. Even a low dose of pure methanol through oral or respiratory exposure might be lethal or result in blindness as a clinical symptom.

## Background

Methanol (CH3OH) is a clear chemical, with a boiling point of 65 °C, a vapor pressure of 96 Torr, and a weak odor; it is a well-known volatile organic compound (VOC). It is used as a solvent for nitrocellulose, ethyl cellulose, various natural or synthetic resins, ethanol denaturant, manufacture of methyl derivative and in daily living environments. It is also a component of coating materials, cleaners (e.g., windshield wiper fluid and car wash liquid), automotive antifreeze, craft adhesives, automotive fuel and combustion, gas and cigarette smoke [1, 2]. Human blood, urine and saliva can contain methanol as a trace element absorbed from the human living environment. Methanol can be easily absorbed during exposure through respiration, skin, and the gastrointestinal tract. The respiratory route is the primary exposure route in workshops and laboratories. At the end of a work shift, pulmonary methanol exposure is approximately 56–82% of the total respiratory intake. After indoor air exposure at a 200 mg/L concentration of methanol, the methanol concentration in the serum reaches the highest value after 4 h of exposure [3].

In human metabolism of methanol, given its physical and chemical properties, high skin absorption is expected. Methanol penetration is predicted at 2.0 mg/cm2/h (logP = −0.77). Skin absorption of methanol vapor is another primary exposure route in terms of respiratory exposure [3]. Of the total exposed methanol in the human body, elimination can occur via exhaling or urination for approximately 10% of unmetabolized methanol from methanol exposure. In the body, 70–80% of the methanol absorbed is metabolized as formaldehyde through alcohol dehydrogenase and then transformed into free radicals and formic acid in the liver and red blood cells, resulting in the final products of carbon dioxide and water through formyl tetrahydrofolate synthetase [1].

The characteristics of methanol poisoning are central nervous system problems, metabolic acidosis, vision loss, and gastrointestinal symptoms [4–6]. Bio-toxicity due to methanol exposure is caused by the metabolites rather than the methanol itself. The severity of the toxicity is related to the degree of metabolic acidosis rather than the concentration of the methanol. Ocular damage might be caused by the toxicity of formic acid from the acidosis of formate. The acidosis is suspected to accelerate the ocular damage [7, 8]. The accumulation of formate fully accounts for the increase in the anion gap and the decrease in plasma bicarbonate. Formate accumulation is the primary or only reason for acidosis in the stages of methanol metabolism [9].

Methanol exposure usually causes acute poisoning rather than chronic poisoning. The main characteristics of acute poisoning are symptoms of metabolic acidosis, ocular toxicity, and nervous system toxicity, and it can cause blindness, narcosis and death, in severe cases. In different species, these toxicities differ in terms of the removal rate of formic acid. If simultaneously exposed to ethanol, it is possible to demonstrate a protective effect on the oxidative competition with alcohols, which can then competitively inhibit the formation of formic acid, as an antagonistic effect of methanol poisoning [10]. As indicated above, methanol poisoning is usually caused by an exposure accident, in most cases through drinking or misuse. Few cases of exposure through the respiration of methanol vapor have been reported [10, 11], such as from the semiconductor cleaning process or methanol poisoning of workers through the manufacturing of mobile phone ports (CNC process). In these workers, symptoms of blindness, vomiting, mental fog, and optic nerve damage are the typical symptoms of methanol poisoning that have been confirmed from exposure to high concentrations of methanol vapor [12, 13].

Recently, methanol exposure from the domestic manufacturing industry has become a major health focus in Korea, for the general population and hazardous material workers. However, few reports have performed systemic evaluations of the clear exposure sources for methanol or examined the exposure level and dose-response relationship between methanol exposure and toxicities in the human body. Therefore, this literature review investigated the relationship between the concentration of methanol exposure and the clinical symptoms and biological exposure markers.

## Methods

Research reports of methanol exposure published in MEDLINE and RISS from 1980 to 2016 were examined in this study. To investigate methanol exposure, the clinical symptoms, toxicities and biological monitoring data from a total of 1768 papers were identified in searches using the keywords ‘methanol’ and ‘exposure’. Among the papers, 29 papers from MEDLINE (from SCI grade medical science journals) and 3 papers in RISS (for Korean journals) were available articles for this study after peer review. The 32 available papers (29 from MEDLINE and 3 from RISS) were examined for data on the exposure source, route and toxicities and biological monitoring of human exposure. Other references included reports from international and domestic authorized public institutions. From the published articles, the human lethal methanol doses were collected, and compared to the data; the, the range of human lethal doses and the median were estimated. From the relationship between the biological exposure indices and exposure concentration levels of methanol, correlation and regression equations and clinical symptoms for methanol exposure levels were recalculated.

## Results and discussion

### Dietary and respiratory intake of methanol

Methanol exposure in the general population primarily occurs through oral intake of meat or vegetables. Fruit and vegetable juice (average 140 mg/L, range 12–640 mg/L), fermented beverages (1500 mg/L or more), and beverages such as soft drinks also provide a considerable amount of methanol. In addition, local traditional liquor, Korean wine, whiskey, and vodka contain a considerable amount of methanol [10]. Smoking a cigarette provides methanol during the vapor phase (approximately 180 μg methanol). Approximately 10% of aspartame, an artificial sweetener, is converted (when hydrolyzed) into methanol in the human body [1].

Background monitoring of a Japanese population revealed that the concentration of methanol in the urine is less than 2 mg/L in most cases and less than 5 mg/L at the 95th percentile [14]. The arithmetic mean value of methanol in urine has been reported as 1.9 mg/L, and the geometric mean value has been reported as 1.7 mg/L [14]. The following values for urinary methanol have been reported in Brazil: 2.26 ± 1.26 mg/L (the arithmetic mean and standard deviation), 2.10 mg/L (the geometric mean), with a range from 0.50–4.78 mg/L [15]. In South Korea, in terms of the exposure concentration and biological monitoring of the general population, few reports are available, unfortunately. It is possible that not more than 2 mg/L of methanol is found in urine, in alignment with the Japanese and Brazilian reports.

### The relation between ambient/indoor air and biological exposure indices of methanol

Linear regression analysis of urinary methanol levels at the end of a work shift revealed a methanol concentration of 5500 mg/L of in ambient air [14]. In another study, there was a correlation between blood and urinary methanol concentrations of 500 mg/L and 1100 mg/L (650 and 1430 mg/m^3^), respectively, from methanol vapor [16]. Table 1 shows the methanol concentration of workroom air and urinary concentrations. The urinary methanol level in male employees was 21.8 ± 20.0 mg/L on average (19.2 mg/L, median and 0.6–57.3 mg/L, range) in exposed groups at the end of a work shift and 1.1 ± 0.9 mg/L on average (0.6–2.9 mg/L, range) in a non-exposed group. It has been estimated that a median urinary methanol concentration of 19.2 mg/L corresponds to a breathing concentration geometric mean of 93 mg/L in the workers’ atmosphere. In reports of exposure to methanol concentrations of 200 mg/L for 8 h, approximate levels of 40 mg/L [17], 42 mg/L (Kawai et al.), and 26 mg/L (arithmetic mean, Yasugi et al.) have been reported for urinary methanol concentrations correlated with workroom air concentrations [18].

**Table 1:** Exposure dose and effects of urinary biological indices of methanol

Methanol in respiratory air (mg/L)	Urinary methanol concentration (mg/L)	References
Occupational exposure		
66.6	23	[18]
70	21.8	[17]
152	40	[17]
181.4	74	[37]
200	40	[17]
200	42	[14]
200	26	[18]
Background exposure		
0.06–0.32 (μg/l)	0.73	[1]
-	1.1	[17] (as control group)

The exposure period was 8 h following the end of a work shift for occupational exposure and 24-h exposure for environmental exposure

As shown in Table 1, methanol exposure doses from ambient air and the response to urinary methanol concentrations could allow for the following regression equation. The regression equation showed 0.21117 for a and 4.69191 for b for Y = aX + b. Pearson’s correlation coefficient was 0.792. The equation showed a linearity between the concentration of methanol in the atmosphere (X) and the urinary methanol concentration (Y). The equation confirmed a dose-response relationship between the inhalation level (X) and urinary concentration of methanol (Y) [19].

### Human lethal dose of methanol

The lethal dose of methanol in human beings has not yet been clearly reported (Table 2). If medically untreated, the lethal dose was approximately 1000 mg/kg (oral intake) if there was no simultaneous ingestion of ethanol. Serious intoxication appeared when the blood formate level was 500 mg/L or more for 10 h of exposure. In an IPCS report, the human lethal dose through oral ingestion is approximately 300–1000 mg/kg [1]. In the OHS report, the lethal dose was reported as 60 ~ 240 ml in a case where a human drank pure methanol. The lowest lethal dose in the report was 10 mg/kg for women, in some cases [20]. In case reports, acute methanol poisoning (60 ~ 600 ml of pure methanol) caused acute metabolic acidosis in 28 young men in Papua New Guinea in 1977. These young men had serious visual impairment and acute pancreatitis 8–36 h after drinking methanol. Four of them died within 72 h after admission, but 16 of the 24 people did not experience symptoms, six were visually impaired, two people demonstrated speech defects and visual impairment [1]. According to the autopsy reports, 6 people died with an 840–5430 mg/L blood methanol concentration and a formic acid concentration of 640 ~ 1100 mg/L [21]. Therefore, using 300 mg/kg, which has been reported as the minimum lethal dose, and assuming the average Korean weight of 65 kg, then 19,500 mg can be calculated as the lethal dose. If a lethal dose of 1000 mg/kg is used, as previously reported, a lethal dose of 65,000 mg can be calculated. Therefore, the lethal dose ranges from 19,500–65,000 mg in humans. In grams (g), the final volume can be estimated as 19.5–65 g per person for a lethal dose. Mass is multiplied by 0.79 for the specific gravity of methanol (calculated as 15.8–189.6 g), and 47.4–474 g/per person is the lethal dose range (based on reports of 20–240 ml from OHS MSDS and 60–600 ml from IPCS), therefore, a small amount of methanol taken orally might be a lethal dose. Therefore, the median lethal dose is estimated as a representative value (56.2 g/per person) based on data using the lethal dose of pure methanol.

**Table 2:** Lethal dose of methanol

Dose	Estimation from recalculation (g/person)	Remarks
Oral administration		
300–1000 (mg/Kg)	19.5–65	[1]
1000 (mg/kg)	65	[22]
20–240 (ml)	15.8–189.6	OHS MSDS
60–600 (ml)	47.4–474	[1]
Respiratory administration		
4000–13,000 mg/L		[22], 12 h exposure

In case reports of respiratory exposure through vapor, women died at levels of 4000–13,000 mg/L after 12 h of methanol exposure with limited ventilation in a closed space [22].

### Exposure levels of methanol and clinical symptoms

Intoxication by exposure level initially manifests as temporary sickness and drowsiness similar to ethanol poisoning. In most cases, methanol intoxication has a latent period of 6 to 30 h. Then, metabolites from methanol can cause vomiting, dizziness, abdominal pain, diarrhea, difficulty breathing, acidosis (Kussmaul breathing), blurred vision, hyperemia of the optic nerve head, blindness, optic disc congestion, manic excitement, manic disorder, delirium, and brain edema [23, 24].

### Oral exposure level of methanol and clinical symptoms

Permanent blindness has been reported to be induced by oral ingestion at levels of 4 ~ 10 ml or 15 ml. However, even after ingesting 500 to 600 ml of methanol, one patient recovered; individual differences appear to be related to whether there is simultaneous exposure to ethanol [25]. With oral exposure, abdominal discomfort and abdominal colic frequently occur, and nausea, vomiting, and diarrhea appear in approximately 50% of cases. Acute pancreatitis, accompanied by amylase viremia and high amylase urine disease, could occur following abdominal pain [23, 26]. Headache and breathing changes appeared at doses of 429 mg/kg dose (from drinking) [20]. Eyes were affected when the subjects drunk 3429 mg/kg of methanol. Changes in circulation, vomiting and dyspnea appeared when subjects ingested 6422 mg/kg of methanol [20, 27, 28]. If these data were converted to 65 kg body weight, the lethal dose range would be 27.8–417 g/person. In these oral intake cases, the exposure was not to pure methanol but rather ethanol. Reportedly, there is not a great difference between the exposure levels that trigger a lethal dose and clinical symptoms (Table 3).

**Table 3:** Symptoms from oral intake of methanol

Exposure level	Estimation from recalculation (g/person)	Symptoms	Remarks
429 mg/kg	27.8	Headache and respiratory change	[20]
3429 mg/kg	222.9	Affects vision	[27]
6422 mg/kg	417	Breathing difficulty, nausea, vomiting, circulatory system changes	[28]
4–15 ml	3.16–11.85	Blindness	[20]

### Blood methanol and its metabolite concentration and clinical symptoms

In patients who were treated in the intensive care unit for serious methanol poisoning, one study reported using hemodialysis treatment in cases of >200 mg/L of blood methanol concentrations from deliberate drinking; the study also reported metabolic acidosis and vision abnormalities [29]. Permanent blindness appeared when the formate blood concentration was 3220 mg/L for 20 h or more from the inhibition of cytochrome oxidase (cytochrome oxidase) in the mitochondria and oxidative phosphorylation process [22]. In a case report of a 4-year-old girl who was hospitalized with nausea, vomiting and abdominal pain in the emergency room, her blood methanol concentrations was 790 mg/L [30]. In a 1978 study of Swedish alcoholism, a cleaning liquid containing up to 80% methanol was ingested. Since 1978, the cleaning liquid methanol has reportedly limit the amount of methanol to less than 5%. The misuse of cleaning solution for alcohol intoxication has been frequently reported, and the methanol concentration in the blood ranged from 1000 to 2000 mg/ from 1 to 2 weeks of exposure [31]. Women 26 years of age undergoing pregnancy care at gestational week 38 consumed 250–500 ml of methanol. Five hours after ingestion, they demonstrated light acidosis, 2300 mg/L of serum methanol, and 336 mg/L of formic acid concentration. Frenia and Schauben [32] have reported toluene (43.8%), methanol (22.3%), methylene chloride (20.5%) and propane (12.5%) in the vapor of a liquid carburetor cleaner. Blood methanol levels from inhalation exposure ranged from 504 to 1286 mg/L, and blood formic acid levels of 120, 193, and 480 μg/ml resulted in altered vision according to eye examinations [32].

### Respiration exposure concentration and clinical symptoms

Amblyopia from vapor inhalation in occupational exposure to methanol (visually impaired) and approximately 100 deaths were reported until 1912 [1, 33]. Most patients had been exposed to methanol vapor (300 mg/L) [34, 35]. Wood heel manufacturing industry workers were exposed to a concentration of methanol vapor ranging from 160 to 780 mg/L. However, the workers did not show any health symptoms [22]. Workers repeatedly exposed to 200–375 mg/L methanol vapor report recurrent headaches [27]. Chronic intoxication occurs when individuals are exposed to a concentration of 800 ~ 3000 mg/L in ambient air through inhalation from occupational exposure [16]. In the case of chronic intoxication through inhalation, it is possible to experience the first signs of visual impairment. For instance, light blurred vision, diminished visual field and permanent blindness could occur. In repeated or long-term respiration exposure, clinical symptoms can occur that are similar to those reported in acute toxicity cases from oral intake. The signs and symptoms from repeated exposure at levels of 365–3080 mg/L were headache, dizziness, vomiting, severe upper abdominal pain, low back pain, breathing difficulties, cloudy view and diarrhea [22]. In a patient who inhaled 3000 mg/L of methanol, bradycardia, visual changes and headache were reported [20]. Workers exposed to methanol concentrations of 1200 ~ 8000 mg/L for 4 years demonstrated diminished visual field and liver expansion [20]. The 23 workers exposed to a poor working environment (power failure prevention work during wartime) at a concentration of approximately 8300 mg/L showed symptoms of temporary blindness (Table 4) [22].

**Table 4:** Respiratory intake symptoms and exposure limit level of methanol

Exposure level (mg/L)	Symptoms	Remarks
160–780	Health symptoms	[22]
200–375	Recurrent headaches	[27]
200	–	[38]
250	–	[38]
228.5–417.7	Optic neuritis, Unconsciousness, Vomiting	[36]
300	Amblyopia	[34]; [35]
365–3080	Blurred vision, Headache, Dizziness, Nausea, Shortness of breath, Severe upper abdominal pain, Back pain, Difficulty breathing, Diarrhea, Bradycardia	[22]
800–3000	Chronic intoxication	[16]
1103–2220	Blindness, Optic neuritis, Loss of pupillary reflex, Amblyopia, Metabolic encephalopathy, Vomiting, Unconsciousness	[36]
3000	Visual changes, Headache, Respiratory changes	[20]
1200–8000	Symptoms of reduced vision, Chronic poisoning	[20]
8300	Temporary blindness	[22]

In a recent methanol addiction accident in Korea, close attention should be directed toward the exposure level of methanol and the initial exposure concentration range. Symptoms of optic neuritis were supposed to appear at an initial concentration of 228.5 mg/L; however, a methanol exposure concentration range of 228.5 to 417.7 mg/L is a somewhat narrow range. Blindness was supposed to occur at an initial exposure concentration of 1103 mg/L, ranging from 1103 to 2220 mg/L, according to the Korean Confederation of Trade Unions, 2016. These initial exposure concentrations for optic neuritis and blindness were the lowest exposure concentrations reported in recent studies of methanol exposure [36].

In 2016, the methanol exposure limit for workers in ACGIH were shown to be 200 mg/L for TWA and 250 mg/L for STEL. These exposure limit levels were thought to be high because health symptoms can appear at 160 mg/L, recurrent headaches at 200 mg/L and optic neuritis, unconsciousness and vomiting at 228.5 mg/L for the initial exposure concentration (Table 4) [36].

### Skin exposure and clinical symptoms

Toxicity through the skin occurs in animals and humans. When the skin comes in contact with methanol liquid, there is a possibility of skin irritation and skin absorption [1, 22]. Metabolic acidosis can result in adverse effects in the eyes and central nervous system. Chronic and repeated continuous contact with methanol can cause erythema, degreasing of the skin, squama, eczema, and dermatitis. For chronic absorption, metabolic acidosis can demonstrate the same symptoms described for acute ingestion [22]. Repeated prolonged contact can cause conjunctivitis, which accumulates a high concentration of methanol when exposed daily to methanol vapor [1].

## Conclusion

From the reported data from methanol exposure in human beings, the dose response is related to methanol exposure dose and urinary methanol concentration. The relationship between the level of oral/respiration exposure and clinical symptoms was reviewed in previous reports. The lethal dose of pure methanol in humans is estimated at 15.8–474 g/person as the range and 56.2 g/person as the median. Oral intake of 3.16–11.85 g/person of pure methanol could cause blindness. Even at low dose levels, pure methanol (oral and respiration exposure) might result in a lethal dose or result in blindness as a clinical symptom. Careful attention is necessary.
